# The Abnormal Glycopatterns of Salivary Glycoproteins in Esophageal Squamous Cell Carcinoma Patients

**DOI:** 10.3389/fchem.2021.637730

**Published:** 2021-03-04

**Authors:** Jian Shu, Jun Ma, Xiameng Ren, Jian Wang, Yan Wang, Kun Zhang, Hanjie Yu, Xiangqian Guo, Zheng Li

**Affiliations:** ^1^ Laboratory for Functional Glycomics, College of Life Sciences, Northwest University, Xi'an, China; ^2^ Institute of Digestive Disease of Zhengzhou University, Zhengzhou, China; ^3^ Department of Clinical Laboratory, The Second Affiliated Hospital of Zhengzhou University, Zhengzhou, China; ^4^ Department of Oncology, The Second Affiliated Hospital of Zhengzhou University, Zhengzhou, China; ^5^ Institute of Biomedical Informatics, Cell Signal Transduction Laboratory, Bioinformatics Center, Henan Provincial Engineering Center for Tumor Molecular Medicine, School of Basic Medical Sciences, Henan University, Kaifeng, China

**Keywords:** esophageal squamous cell carcinoma, protein glycosylation, saliva, lectin microarrays, MALDI-TOF/TOF-MS

## Abstract

Glycosylation is one of the most crucial posttranslational modifications of proteins, containing a remarkable amount of biological information. The alteration of glycosylation is closely associated with certain diseases. Exploring glyco-code in the development of diseases is a hot topic in recent years. Esophageal squamous cell carcinoma (ESCC) is the primary pathological histology in developing countries and a severe threat to human health. Although the glycan profiles in the blood samples of ESCC patients were analyzed using glycomic and glycoproteomic methods, the difference of salivary glycopatterns between healthy subjects and ESCC patients is not explicit yet. In the present study, ESCC patients (n = 16) and healthy volunteers (HVs, n = 25) were enrolled. The glycomic strategy combining lectin microarray and lectin blotting was employed to investigate and confirm the altered salivary glycopatterns. Datura stramonium (DSA) was selected to isolate the GlcNAc or Galβ1-4GlcNA-containing glycoproteins due to the distinct difference between ESCC patients and HVs. The N-glycans from DSA-enriched glycoproteins were released by PNGase F and further identified by MALDI-TOF/TOF-MS to obtain the precise structural information of the altered glycans. As a result, the glycopatterns recognized by 13 lectins (e.g., ECA, RCA120, and DSA) showed significant alterations in ESCC patients’ saliva. The ESCC patients showed higher levels of GalNAc and Gal, sialic acid, and GlcNAc expression profiles and lower levels of mannose and fucose expression profiles. The MALDI-TOF/TOF-MS results indicated that the proportion of the GlcNAc or Galβ1-4GlcNAc-containing N-glycans was increased in ESCC patients (79.04%) compared with HV (63.20%), which was consistent with the results of lectin microarrays. Our findings provide comprehensive information to understand the complex physiological changes in ESCC patients. And the altered salivary glycopatterns such as GlcNAc or Galβ1-4GlcNAc-containing N-glycans recognized by DSA might serve as potential biomarkers for the diagnosis of ESCC patients.

## Introduction

Esophageal cancer (EC) ranks as the seventh most prevalent cancer with more than 570 thousand new cases and the sixth most lethal cancer with over 500 thousand deaths worldwide (Bray et al., 2018). EC is also a common type of malignant cancer in China, ranking fourth in terms of diagnosis and death (Chen et al., 2016). China is even responsible for more than 50% of the world’s EC new cases because of the large population size and high incidence. As one of the major histologic types of EC, esophageal squamous cell carcinoma (ESCC) is still accounted for the vast majority of EC in developing countries, while the other crucial histologic kind of EC, esophageal adenocarcinoma, has become the predominant type in developed countries, and the incidence rate continues to rise (Malhotra et al., 2017). ESCC is a serious threat to human health, is distributed anywhere in the esophagus, and is considered positively associated with heavy alcohol drinking, tobacco smoking, and nutrients lacking. It is reported that more than 50% of ESCC patients were diagnosed only at the advanced stages due to the unspecific symptoms and lack of early biomarkers, resulting in poor prognosis and a low 5-year survival rate (Ohashi et al., 2015; Kojima and Doi, 2017).

The assembly of glycans is complex enzymatic progress catalyzed by a series of glycosyltransferases and glycosidases, which starts in the endoplasmic reticulum and matures in the Golgi apparatus (Chao et al., 2020). As the most prevalent posttranslational modifications, glycosylation occurs on approximately 70% of human proteins and involves a wide range of biological processes, which could partially reflect human's physiological states (Yu et al., 2020a; de Haas et al., 2020). The alterations of glycosylation have been widely detected in various cancers and have shown a profound correlation with carcinogenesis (Oliveira-Ferrer et al., 2017; Verhelst et al., 2020). Overexpressions of sialylation, fucosylation, branched glycans, and truncated O-glycan are considered the most widely occurring cancer-associated glycans changes in tissue and cell samples (Pinho and Reis, 2015). Although altered glycans were detected in human cancer tissue, cells, serum, and urine samples decades ago, investigations in the past few years have demonstrated that altered glycosylation is also present in saliva samples. Compared with the most commonly used diagnostic fluids of blood and urine, saliva offers the advantages of being noninvasive, easy, secure, and cost-effective, which prompts an interest in evaluating its diagnostic utility value (Nunes et al., 2015). Previous studies have demonstrated that different types of diseases (e.g., gastric cancer (GC), type 2 diabetes mellitus (T2DM), hepatopathy, breast disease oral lichen planus, Keshan disease, and oral ulcer) could induce the different alternations of the salivary protein glycopatterns (e.g., fucosylation in GC and sialylation in T2DM) (Zhang et al., 2016; Fang et al., 2017; Wang et al., 2017; Liu et al., 2018; Shu et al., 2018; Yu et al., 2020b). The accumulating data also indicated that the saliva glycopatterns might serve as potential biomarkers to classify cancer cases from controls. The high-throughput glycomic method of lectin microarray could be used to directly analyze the glycosylation of crude samples without the liberation of glycans, providing a global snapshot of glycosylation state in its native context (Hirabayashi et al., 2013; Yu et al., 2020a). As an efficient screening tool, the lectin microarrays usually work together with lectin blotting and mass spectrometry for screening, analysis, and validation of the important glycopatterns of glycoprotein.

In this study, the altered salivary glycopatterns related to ESCC were investigated and confirmed by the integrated glycomics strategy. To acquire precise structural information of the altered glycans between ESCC and healthy volunteers (HV) cases, the glycoproteins were isolated by lectin-mediated affinity capture, and the N-glycans were released and purified from the isolated glycoproteins and then identified using MALDI-TOF/TOF-MS. The purpose of this study is to clarify the altered salivary protein glycopatterns related to ESCC and to identify the precise structures of the altered salivary glycopatterns, which may help us to understand the complex physiological changes in ESCC patients.

## Materials and Methods

### Study Approval and Population

The collection and use of the whole saliva for research in this study were approved by the Ethical Committee of Northwest University (Xi’an, China) and the Second Affiliated Hospital of Zhengzhou University (Zhengzhou, China). Written informed consent was received from participants. And this study was conducted by the ethical guidelines of the Declaration of Helsinki. After a standardized endoscopic procedure and histopathological evaluation, the individuals diagnosed with primary ESCC (n =16) were enrolled in this study. The age- and sex-matched HVs (n = 25) were recruited from the health checkup center in the same hospital where they underwent a medical examination. All the participants had no significant differences between the two groups regarding demographic, socioeconomic, and lifestyle characteristics, and those who received preoperative radiotherapy, chemotherapy, chemoradiotherapy, or antibiotic therapy were excluded from this study. The clinical characteristics of HVs and ESCC patients were summarized in Table 1.

**TABLE 1:** Demographic and clinical information of ESCC patients and HV subjects in this study.

Variables	HVs	ESCC	*p*-value*^a^*
Numbers (n)	25	16	
Age (years)			0.137
≤ 60	11 (44%)	7 (43.75%)	
> 60	14 (56%)	9 (56.25%)	
Gender			0.852
Male	16 (64%)	10 (62.5%)	
Female	9 (37%)	6 (37.5%)	
Pathological (AJCC)^b^			
I-II	—	7 (43.75%)	
III-IV	—	9 (56.25%)	
Differentiation			
Well	—	3 (18.75%)	
Moderate	—	8 (50%)	
Poor	—	5 (31.25%)	

^a^ *p* value was derived from the nonparametric Mann–Whitney test.

^b^ AJCC, American Joint Committee on Cancer staging system (7th edition).

### Whole Saliva Collection and Preparation

The collection protocol has been described in previous literature (Liu et al., 2018; Shu et al., 2018). The collected whole saliva was centrifuged at 10,000 g at 4°C for 15 min to remove insoluble components. And the cocktail of protease inhibitor (Sigma-Aldrich, United States) was added to the collected supernatant according to the manufacturer’s recommendations and lyophilized at −80°C until use.

### Lectin Microarrays and Data Analysis

The lectin microarrays were produced using 37 lectins with different binding preferences covering N- and O-linked glycans (Shu et al., 2017; Yu et al., 2020b). The Cy3-labeled glycoproteins were incubated on the lectin microarray with gentle rotation in the dark (37°C, 3 h). The microarrays were washed with PBST and PBS, respectively, centrifuged dry, and scanned immediately using a Genepix 4000B confocal scanner (Axon Instruments, United States). The generated images were analyzed by Gene pix software (version 6.0, Axon Instruments Inc., Sunnyvale, CA). The unsupervised average hierarchical cluster analysis (HCA) and principal component analysis (PCA) were performed by Expander 6.0 (http://acgt.cs.tau.ac.il/expander/) and Multivariate Statistical Package (United Kingdom), respectively. And the *p* value was derived from the nonparametric Mann–Whitney test using the GraphPad Prism software (Version 7.0, GraphPad Software, Inc., San Diego, CA).

### Lectin Blotting Analysis

The expression levels of glycan structures were analyzed by lectin blotting as described previously (Zhang et al., 2019; Yu et al., 2020b). The pooled salivary protein was separated by 10% SDS-PAGE and transferred onto PVDF membrane (0.22 mm Millipore, Bedford, MA, United States). The PVDF membrane was blocked by the Carbo-Free Blocking Solution (Vector Labs, Burlingame, CA) and incubated with Cy5-labeled DSA, ECA, LCA, PSA, and UEA-I, respectively. The membrane was scanned by the STORM FluorImager (Molecular Dynamics, Sunnyvale, CA, United States) and measured using the ImageJ software (NIH).

### Preparation of DSA-Magnetic Particle Conjugates

The epoxy-coated magnetic particles (2 mg) were rinsed with ethanol and coupling buffer (5 mM NaB_4_O_7_, 180 mM H_3_BO_4_, 150 mM Na^+^, pH 7.4), respectively, and reacted with DSA solution (DSA dissolve in coupling buffer) according to the protocol (Zhang et al., 2019; Yang et al., 2020b). Then, conjugates were washed with a coupling buffer to remove the unbound lectins.

### Selective Isolation of Glycoprotein Fractions from Saliva by DSA-Magnetic Particle Conjugates

The GlcNAc and Galβ1-4GlcNAc-containing glycoproteins were isolated from ESCC patients and HVs using DSA-magnetic particle conjugates as described previously (Zhang et al., 2019; Yang et al., 2020b). Briefly, the pooled salivary protein was diluted with the binding buffer (100 Mm Tris-HCl; 150 mM NaCl; 1 mM CaCl_2_, MgCl_2_, and MnCl_2_; pH 7.4) and incubated with the DSA conjugates (room temperature, 3 h). After 3 h gentle shaking, the conjugates were washed using washing buffer (0.1% Tween-20 in binding buffer, pH 7.2) to remove unbound proteins, and the glycoproteins bound to the conjugates were eluted with an eluting buffer (8 M urea, 40 mM NH_4_HCO_3_).

### Isolation and Purification of N-Linked Glycans

The isolation and purification of N-glycans were performed based on previously described methods (Qin et al., 2017; Yang et al., 2020b). The glycoproteins were concentrated and desalted by Amicon Ultra-0.5 3 KDa ultrafiltration units (Millipore, United States) and then denatured by the addition of 8 M urea, 10 mM DTT, and 10 mM IAM. The denatured glycoproteins were exchanged to 40 mM NH_4_HCO_3_ buffer and incubated with trypsin (37°C, overnight). The trypsin in the mixture was inactivated by heating (80°C, 5 min) and then the PNGase F (New England Biolabs, Beverly, MA) was added to release the N-glycans (37°C, overnight). Subsequently, the digest was subjected to HyperSep Hypercarb SPE cartridges (25 mg, 1 mL; Thermo Scientific) to remove peptides. The purified N-glycans were collected and lyophilized.

### Characterization of N-Glycans by MALDI-TOF/TOF-MS

The N-glycans were characterized by matrix-assisted laser desorption ionization time-of-flight/time-of-flight mass spectroscopy (MALDI-TOF/TOF-MS, UltrafleXtreme, Bruker Daltonics; Bremen, Germany) as described previously (Qin et al., 2017; Zhang et al., 2019; Yang et al., 2020b). Glycans were resuspended and spotted onto an MTP AnchorChip sample target. Then, 2 μL of 10 mg/mL 2,5-dihydroxybenzoic acid (DHB) with 1 mM NaCl in 50% (v/v) methanol solution was spotted to recrystallize the N-glycans and vacuum dried for analysis. Peptide calibration standards (250 calibration points; Bruker) were used as mass calibration. Positive ion reflection mode was performed, and a mass range of 1,000–4,000 Da was analyzed. Representative MS spectra of N-glycan mass peaks with signal-to-noise ratio above three were generated and annotated using FlexAnalysis and GlycoWorkbench software.

## Results

### Overall Salivary Protein Glycopatterns in Esophageal Squamous Cell Carcinoma Patients

**FIGURE 1:**
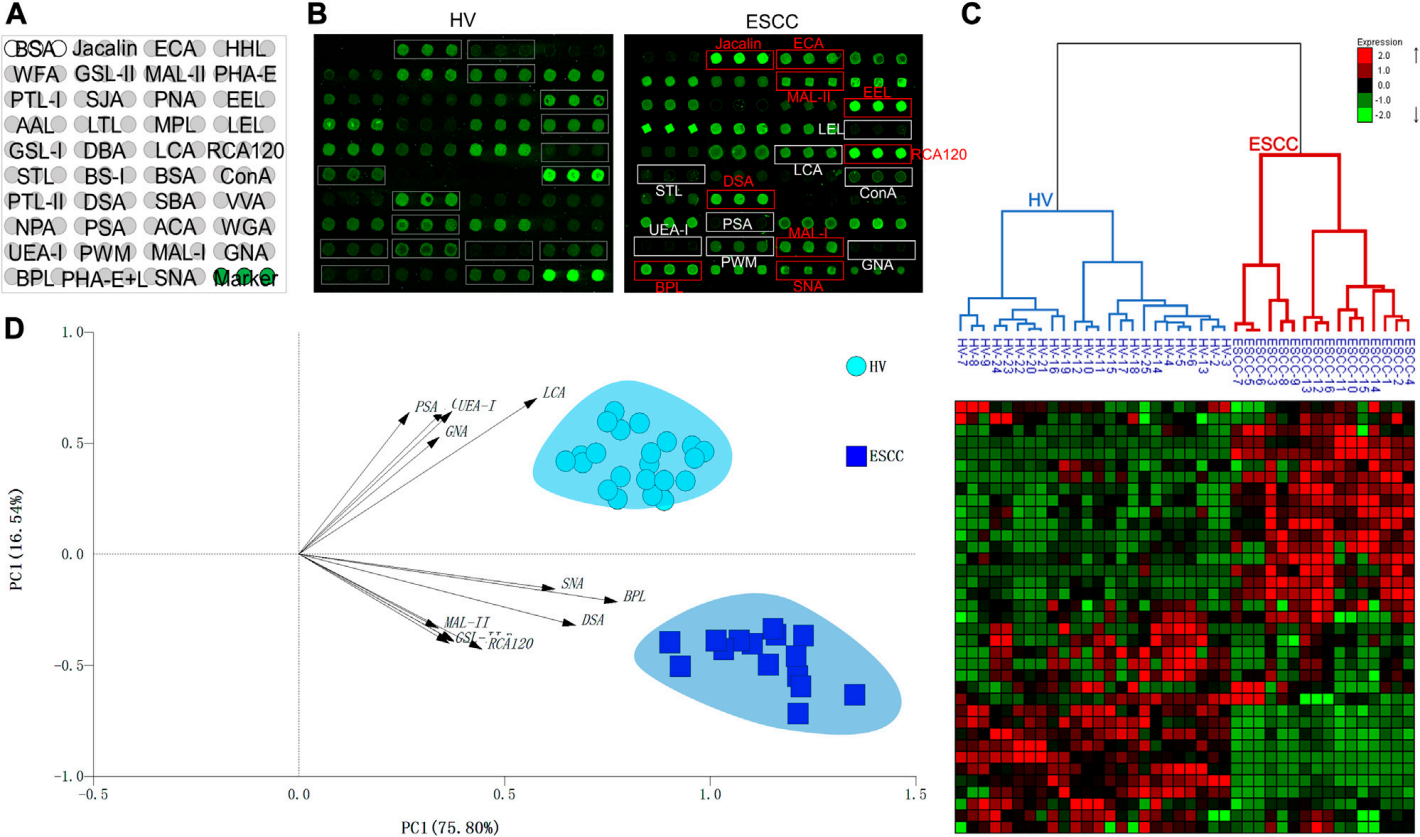
The salivary glycopatterns of HVs and ESCC patients. **(A)** The layout of the lectin microarray. A total of 37 lectins were dissolved in the recommended buffer and spotted on lectin microarray; each lectin was spotted in triplicate per block. **(B)** Glycopattern of HVs **(left)** and ESCC patients **(right)** detected by the lectin microarray. The lectins with increased NFIs in ESCC patients are marked with red boxes, and those with decreased NFIs are marked with white boxes. **(C)** Unsupervised hierarchical clustering analysis of lectins and all samples. The samples were listed in columns, and the lectins were listed in rows. HVs and ESCC patients were clustered based on the correlation similarity of salivary glycopatterns. The color and intensity of each square indicated expression levels relative to other data in the row. Red, high; green, low; black, medium. **(D)** Principal component analysis of 37 lectins for all samples. HV and ESCC samples were indicated by azure circles and blue squares, respectively. PC1: principal components 1. PC2: principal components 2.

A total of 16 ESCC and 25 HV samples were detected by lectin microarrays independently to investigate the altered salivary protein glycopatterns associated with ESCC patients. The layout of the lectin microarrays and typical representative images were listed in Figure 1A,B. HCA and PCA were executed to evaluate the glycan expression profiles of ESCC and HV cases and to provide a graphical representation of relationships among the subjects and diseases. The normalized fluorescent intensities (NFIs) for each lectin were distributed in the heat map by unsupervised clustering method to achieve the hierarchical relationship of the samples based on similarities in their glycan expression pattern. As shown in Figure 1C, 16 ESCC cases were classified into one category, and 25 HV cases were classified into another class, indicating that the salivary glycopatterns identified by these lectins were different between HVs and ESCC patients. A similar result was obtained by PCA as well; the subjects clustered separately by principal components 1 and 2 to form HV and ESCC clusters with different colors and symbols in Figure 1D, representative of the salivary glycopatterns differences for the two groups in a manner.

### Alterations of Salivary Protein Glycopatterns in Esophageal Squamous Cell Carcinoma Patients

**FIGURE 2:**
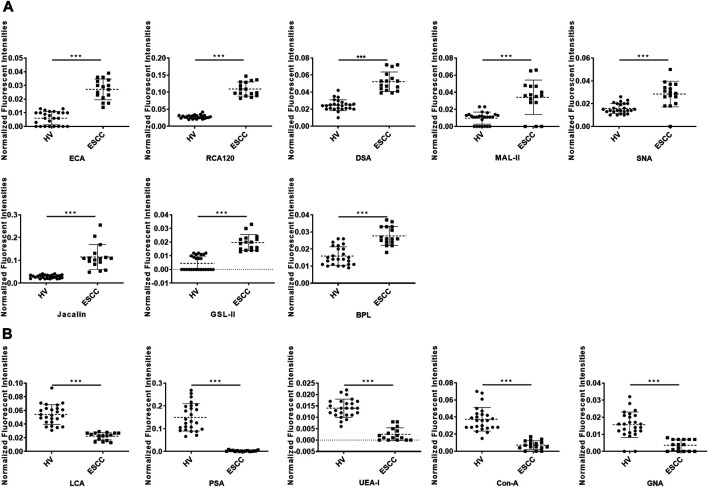
Alterations of salivary glycopatterns in ESCC patients compared to HVs. **(A)** The NFIs of eight lectins were significantly increased in ESCC patients compared to HVs. **(B)** The NFIs of five lectins were significantly decreased in ESCC patients compared to HVs. *p < 0.05, **p < 0.01, and ***p < 0.001.

To further investigate the alteration of salivary protein glycopatterns in ESCC patients, the Mann–Whitney test was used to compare the variance between ESCC and HV groups. In total, 13 lectins exhibited significantly altered NFIs in ESCC cases. As shown in Figure 2 and Table 2, the Galβ1-3/4GlcNAc binder LEL and MAL-I, Galα1-3GalNAc binder BPL, T antigen and sialyl-T antigen binder Jacalin, Siaα2-3/6Gal binder MAL-II/SNA, GlcNAc and Galβ1-4GlcNAc binder DSA, and GSL-II showed increased NFIs in ESCC salivary glycoproteins against HV cases. On the contrary, the high-mannose binder of ConA and GNA, Fucα-1,6GlcNAc binder LCA and PSA, and Fucα1-2Galβ1-4Glc (NAc) binder UEA-I showed decreased NFIs in ESCC salivary glycoproteins compared with HV cases. As a result, ESCC patients showed higher levels of GalNAc and Gal expression profile, sialic acid expression profile, GlcNAc expression profile, and lower levels of mannose expression profile and fucose expression profile.

**TABLE 2:** Altered glycopatterns of salivary glycoproteins between HVs and ESCC patients.

Monosaccharide specificity	Lectin	Preferred glycan structure (terminal epitope)	ESCC
Gal	ECA	Galβ1-4GlcNAc (type II), Galβ1-3GlcNAc (type I)	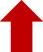
	RCA120	Galβ1-4GlcNAc (type II), Galβ1-3GlcNAc (type I)	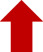
	BPL	Galα1-3GalNAc, GalNAc	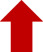
	Jacalin	Galβ1-3GalNAcα-Ser/Thr(T), sialyl-T(ST)	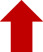
Sialic acid	MAL-II	Siaα2-3Gal	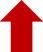
	SNA	Siaα2-6Gal	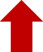
GlcNAc	DSA	GlcNAc, Galβ1-4GlcNAc	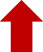
	GSL-II	GlcNAc, agalactosylated tri/tetra antennary glycans	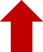
Mannose	ConA	High-mannose, Manα1-6(Manα1-3)Man	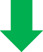
	GNA	High-mannose, Manα1-3Man	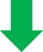
	LCA	Fucα1-6GlcNAc, high-mannose	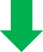
	PSA	Fucα1-6GlcNAc, high-mannose	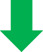
Fucose	LCA	Fucα-1,6GlcNAc, high-mannose	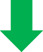
	PSA	Fucα-1,6GlcNAc, high-mannose	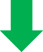
	UEA-I	Fucα1-2Galβ1-4Glc(NAc)	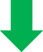

### Validation of Different Glycopatterns Between ESCC Patients and HV Cases

**FIGURE 3:**
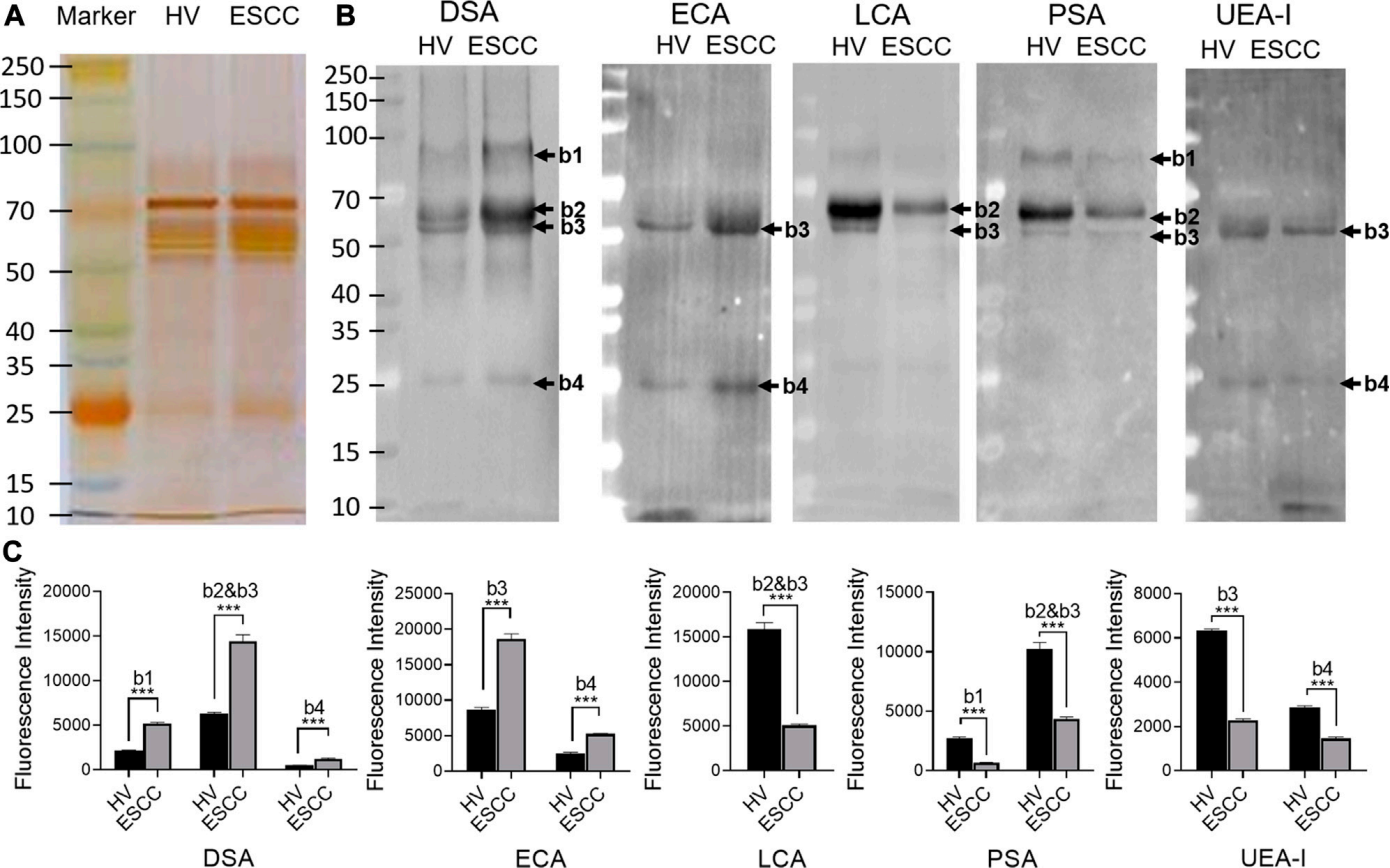
Validation of the differential expressions of the glycopatterns in pooled saliva from HVs and ESCC patients. **(A)** Silver nitrate staining of the salivary proteins from HV and ESCC groups. **(B)** The binding pattern of glycoproteins from ESCC patients and HVs using Cy five labeled lectins of DSA, ECA, LCA, PSA, and UEA-I. The molecular weights of band 1(b1), b2, b3, and b4 were approximately 90, 55, 60, and 25 kDa. **(C)** The gray value of each apparent difference bands was measured by ImageJ software.

SDS-PAGE and lectin blotting analysis were performed with silver staining and Cy5-labeled lectin staining to confirm the different abundances of glycopatterns in pooled saliva from ESCC and HV cases. The results of SDS-PAGE demonstrated that the distribution and abundance of salivary protein bands were similar in ESCC and HV cases (Figure 3A). The result of the lectin blotting analysis showed that the obviously different bands range from 30 to 100 kDa (Figure 3B). The GlcNAc and Galβ1-4GlcNAc binder DSA showed a distinctly increased binding to four apparent bands with molecular weights of approximately 90 kDa (b1), 55 kDa (b2), 60 kDa (b3), and 25 kDa (b4) in the ESCC patients compared with HVs, and Galβ-1,3/4GlcNAc binder ECA staining showed stronger binding intensity to two apparent bands (b3 and b4) in the ESCC patients. On the contrary, Fucα-1,6GlcNAc binder LCA and PSA and Fucα1-2Galβ1-4Glc (NAc) binder UEA-I showed weaker binding to b1, b2, b3, or b4 in ESCC than in HV (Figure 3B,C). The relative binding intensity of these randomly selected lectins to pooled saliva samples was almost coincident with the results from the lectin microarrays.

### The N-Linked Glycan Profiles of the DSA-Isolated Salivary Glycoprotein

**FIGURE 4:**
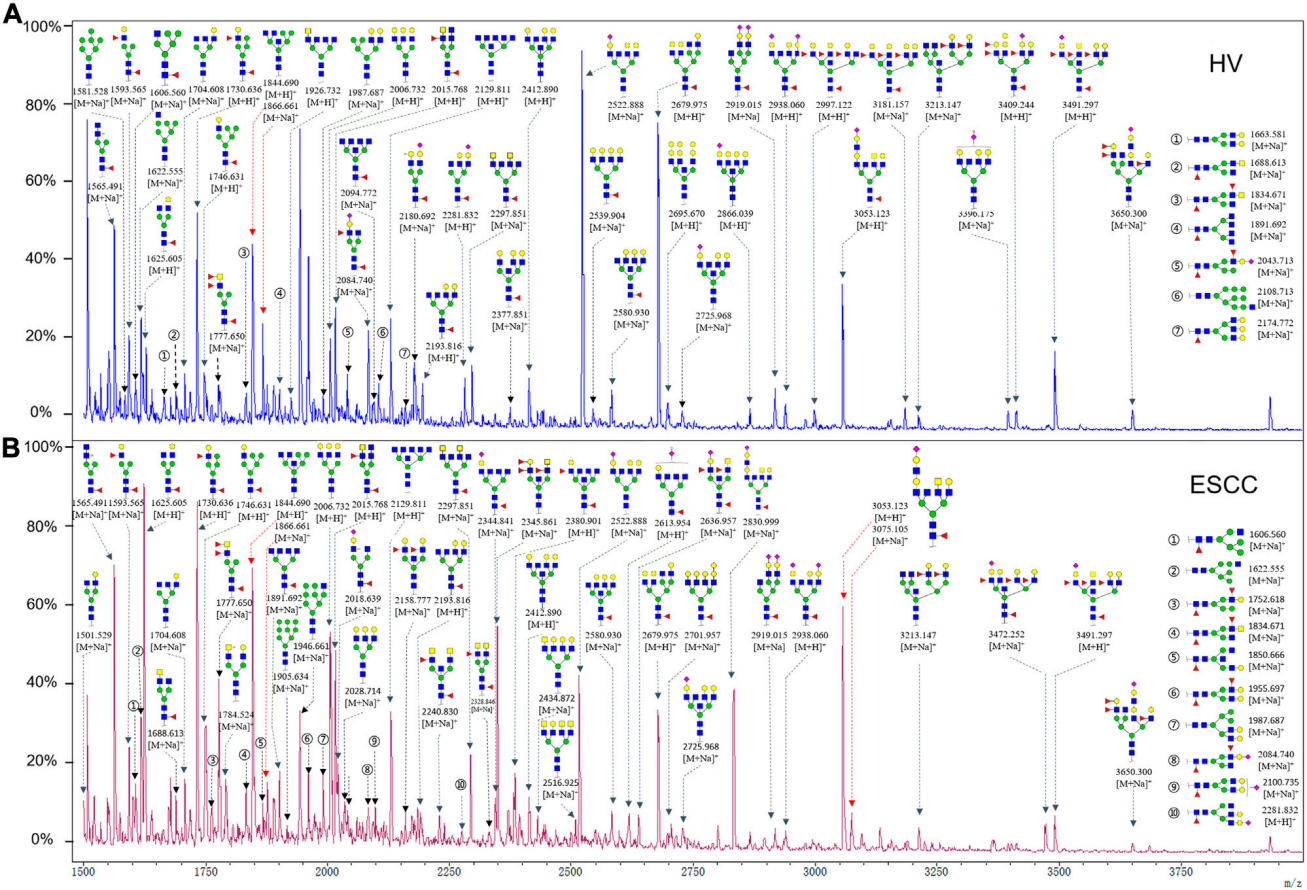
MALDI-TOF/TOF-MS spectra of the purified N-glycans from the DSA-enriched salivary glycoproteins of HVs **(A)** and ESCC patients **(B)**, respectively. Detailed glycan structures were analyzed using the GlycoWorkbench software. Proposed structures and their m/z values were shown for each peak. Blue square, GlcNAc; green circle, Man; yellow circle, Gal; yellow square, GalNAc; purple diamond, NeuAc; red triangle, Fuc.

**FIGURE 5:**
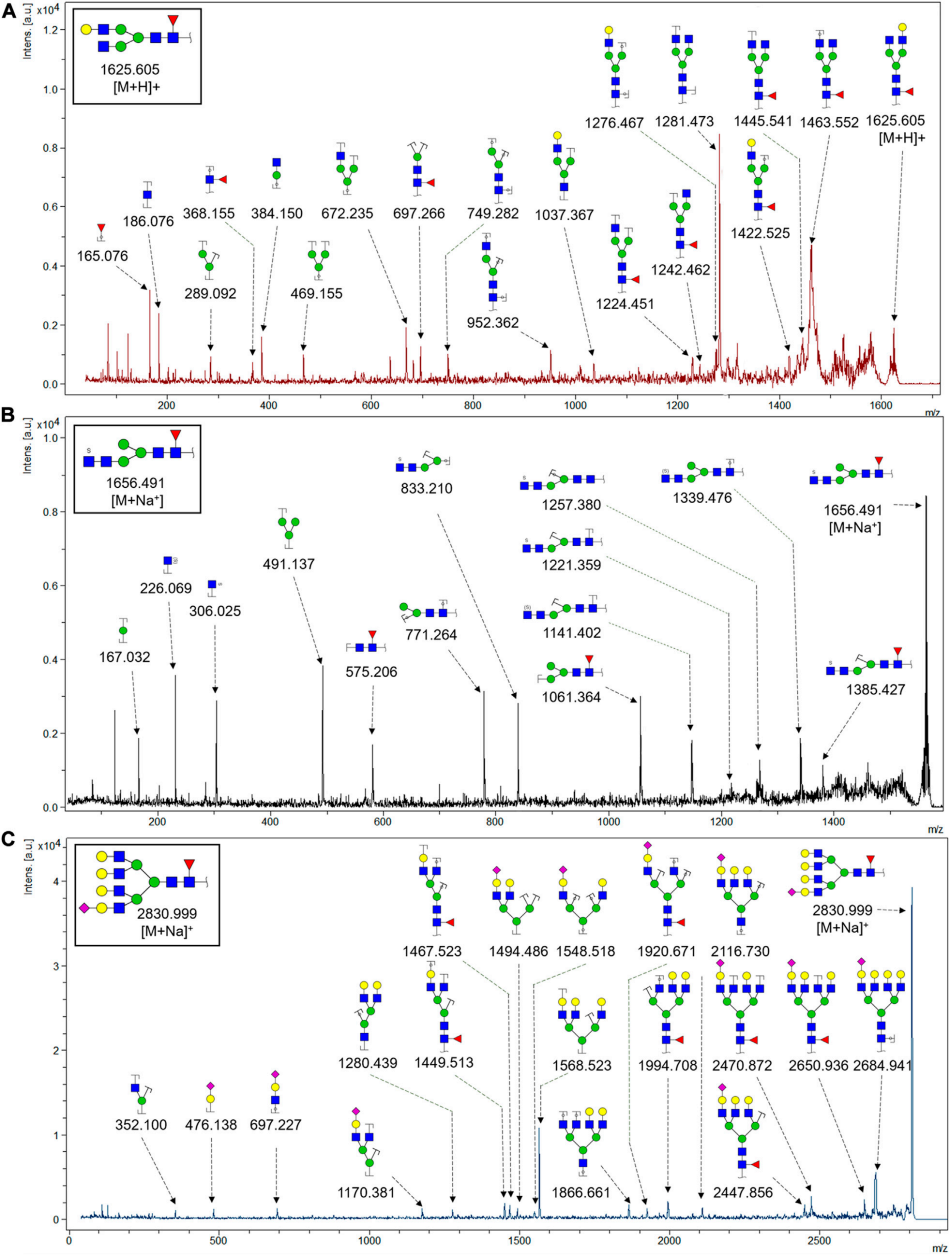
MALDI-TOF/TOF MS/MS analyzing the N-glycan precursor ion from MS spectra. For example, the three N-glycan peaks **(A)** m/z 1625.605, **(B)** 1656.491, and **(C)** 2830.999 were subjected to MS/MS analysis.

To obtain the GlcNAc, Galβ1-4GlcNAc N-glycan structures of glycoproteins in saliva from ESCC and HV cases. The glycoproteins were isolated using the DSA-magnetic particle conjugates, then N- glycans were released by PNGase F, purified by HyperSep Hypercarb SPE cartridges, and identified by MALDI-TOF/TOF-MS, respectively. A total of 48 and 56 N-glycan peaks from the pooled saliva samples of ESCC and HV were identified and annotated with proposed structures in Figure 4A,B. Of these, there were 44 and 52 GlcNAc or Galβ1-4GlcNAc containing N-glycans that could be recognized by DSA to be identified in HV and ESCC, respectively, and their proposed structures were listed in Table 3. It was noticeable that there was an overlap of 31 N-glycans (e.g., m/z 1565.491, 1593.565, and 1625.605) presented in both HV and ESCC cases, while 13 N-glycans (e.g., m/z 1622.555, 1926.732, and 2084.740) were observed only in HV samples and 21 N-glycans (e.g., m/z 1501.529, 1792.624, and 2018.639) detected only in ESCC samples. The increased tendency of GlcNAc or Galβ1-4GlcNAc-containing N-glycans in ESCC samples compared with HVs was presented in the numbers and relative abundance levels. The proportion of the N-glycans with the GlcNAc or Galβ1-4GlcNAc moieties was increased in ESCC (79.04%) compared with HV (63.20%), which was consistent with the results of lectin microarrays. The MS/MS analysis was further performed to determine the exact glycan structures. For example, the MS/MS spectra of the precursor ions m/z 1625.605, 1656.491, and 2830.999 were illustrated in Figure 5.

**TABLE 3:** The GlcNAc, Galβ1-4GlcNAc-containing N-glycan peaks of DSA-isolated salivary glycoproteins in the present study.

NO	Calculated m/z	Experimental mass m/z		Charge	Proposed structure*^a^*	% N-glycans*^b^*	
		HV	ESCC			HV	ESCC
1	1501.529	—	1501.511	[M + Na]^+^	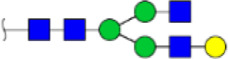	—	1.10
2	1565.491	1565.472	1565.474	[M + Na]^+^	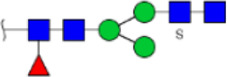	5.30	5.47
3	1593.565	1593.532	1593.545	[M + Na]^+^	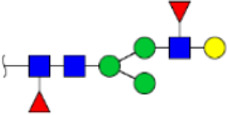	2.26	1.90
4	1606.560	1606.338	1606.341	[M + Na]^+^	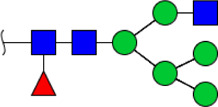	0.91	1.15
5	1622.555	1622.528	1622.533	[M + Na]^+^	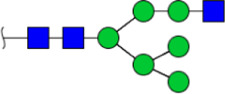	1.19	2.45
6	1625.605	1625.553	1625.563	[M + H]^+^	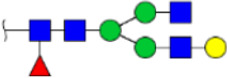	1.65	7.56
7	1663.581	1663.496	—	[M + Na]^+^	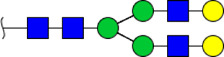	0.59	—
8	1688.613	1688.424	1688.425	[M + Na]^+^	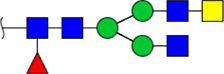	0.59	0.70
9	1704.608	1704.593	1704.593	[M + Na]^+^	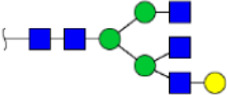	1.09	1.10
10	1730.636	1730.579	1730.606	[M + H]^+^	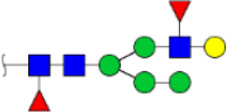	5.99	7.29
11	1746.631	1746.587	1746.592	[M + H]^+^	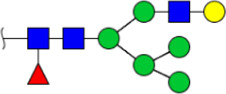	0.92	1.72
12	1752.618	—	1752.608	[M + Na]^+^	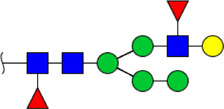	—	0.94
13	1784.565	—	1784.524	[M + Na]^+^	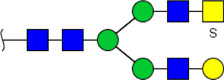	—	1.35
14	1834.671	1834.528	1834.575	[M + Na]^+^	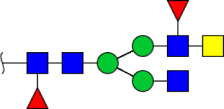	0.73	0.71
15	1844.69	1844.591	1844.593	[M + H]^+^	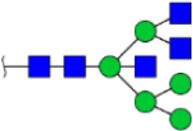	3.96	4.45
	1866.661	1866.558	1866.574	[M + Na]^+^		2.03	0.62
16	1850.666	—	1850.642	[M + Na]^+^	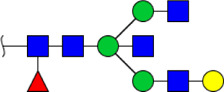	—	0.94
17	1891.692	1891.683	1891.660	[M + Na]^+^	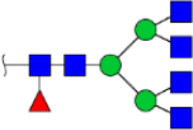	0.66	0.76
18	1926.732	1926.675	—	[M + H]^+^	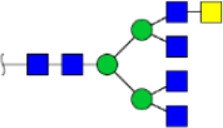	0.66	\
19	1946.661	—	1946.668	[M + Na]^+^	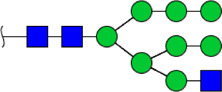	—	2.28
20	1955.697	—	1955.679	[M + Na]^+^	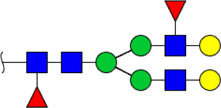	—	1.25
21	1987.687	1987.535	1987.558	[M + Na]^+^	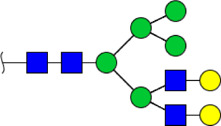	0.69	0.90
22	2006.732	2006.574	2006.546	[M + H]^+^	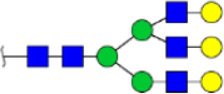	1.60	4.10
23	2015.768	2015.721	2015.725	[M + H]^+^	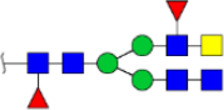	2.28	2.47
24	2018.639	—	2018.568	[M + Na]^+^	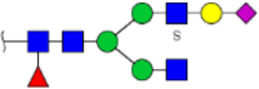	—	0.91
25	2028.714	—	2028.582	[M + Na]^+^	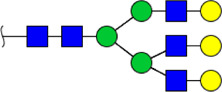	—	0.46
26	2084.740	2084.620	2084.533	[M + Na]^+^	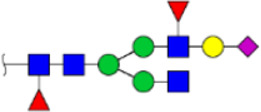	2.15	0.63
27	2094.772	2094.581	—	[M + Na]^+^	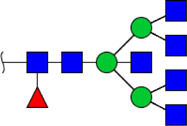	0.56	—
28	2100.735	—	2100.549	[M + Na]^+^	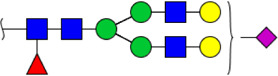	—	0.51
29	2108.713	2108.626	—	[M + Na]^+^	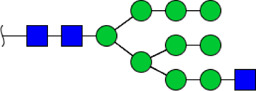	0.79	—
30	2129.811	2129.695	2129.742	[M + H]^+^	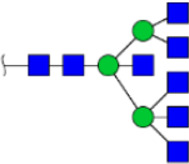	1.90	1.52
31	2158.777	—	2158.768	[M + Na]^+^	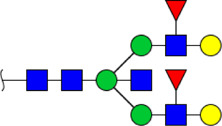	—	0.42
32	2174.772	2174.600	—	[M + Na]^+^	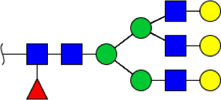	0.53	—
33	2193.816	2193.721	2193.798	[M + H]^+^	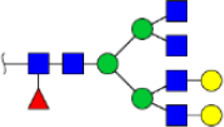	0.88	0.45
34	2240.830	—	2240.745	[M + Na]^+^	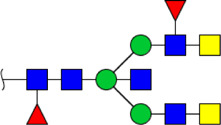	—	0.38
35	2281.832	2281.683	2281.705	[M + H]^+^	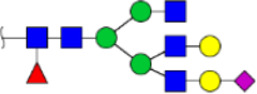	0.99	0.37
36	2297.851	2297.918	2297.956	[M + Na]^+^	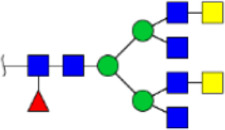	1.05	1.08
37	2344.841	—	2344.843	[M + Na]^+^	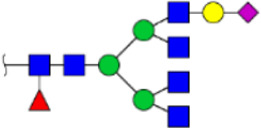	—	3.19
38	2377.851	2377.667	—	[M + Na]^+^	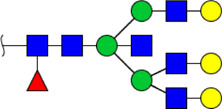	0.32	—
39	2345.861	—	2345.698	[M + Na]^+^	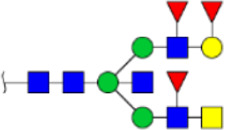	—	3.19
40	2380.901	—	2380.872	[M + H]^+^	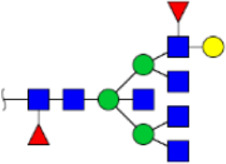	—	1.06
41	2412.890	2412.867	2412.940	[M + H]^+^	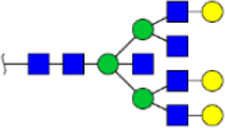	0.88	0.44
42	2434.872	—	2434.812	[M + Na]^+^	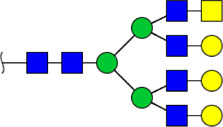	—	0.43
43	2516.925	—	2516.893	[M + Na]^+^	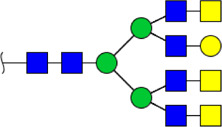	—	0.36
44	2522.888	2522.924	2522.781	[M + Na]^+^	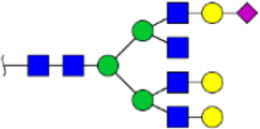	7.23	2.45
45	2539.904	2539.764	—	[M + Na]^+^	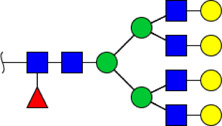	0.31	—
46	2580.930	2580.804	2580.830	[M + Na]^+^	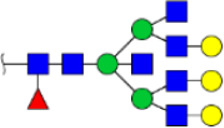	0.53	0.26
47	2613.954	—	2613.930	[M + H]^+^	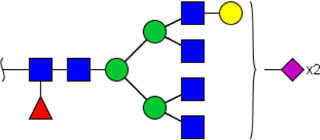	—	0.47
48	2636.957	—	2637.031	[M + Na]^+^	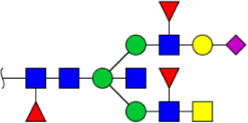	—	0.43
49	2679.975	2679.794	2679.795	[M + H]^+^	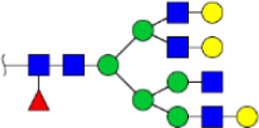	5.52	1.90
50	2695.670	2695.824	—	[M + H]^+^	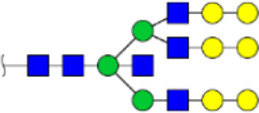	0.38	—
51	2701.957	—	2702.092	[M + Na]^+^	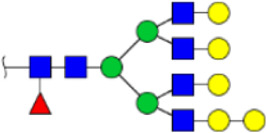	—	0.30
52	2725.968	2726.128	2726.128	[M + Na]^+^	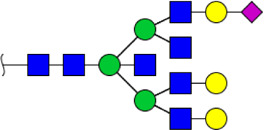	0.20	0.19
53	2830.999	—	2830.999	[M + Na]^+^	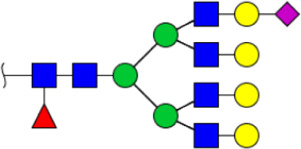	—	1.91
54	2866.039	2866.208	—	[M + H]^+^	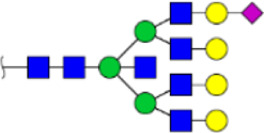	0.26	—
55	2919.015	2918.965	2918.981	[M + Na]^+^	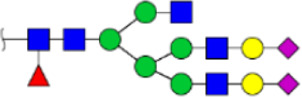	0.66	0.25
56	2938.060	2938.150	2938.126	[M + H]^+^	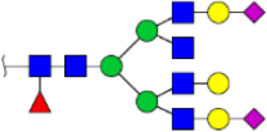	0.37	0.24
57	2997.122	2997.246	—	[M + H]^+^	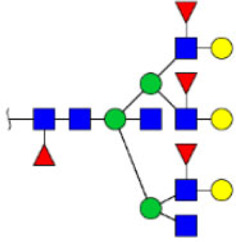	0.32	—
58	3053.123	3053.293	3053.247	[M + H]^+^	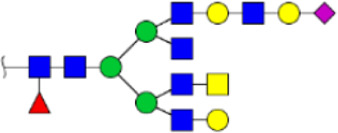	2.18	2.73
	3075.105	—	3075.261	[M + Na]^+^		—	0.42
59	3181.157	3181.005	—	[M + Na]^+^	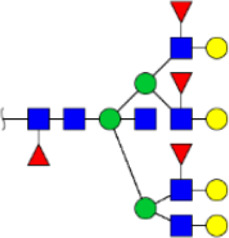	0.28	—
60	3213.147	3213.112	3213.305	[M + Na]^+^	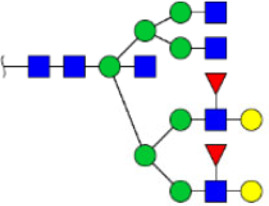	0.21	0.24
61	3396.175	3396.318	—	[M + Na]^+^	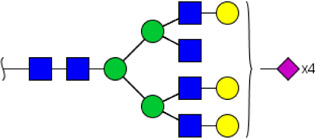	0.24	—
62	3409.244	3409.435	—	[M + H]^+^	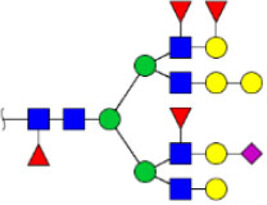	0.21	—
63	3472.252	—	3472.428	[M + Na]^+^	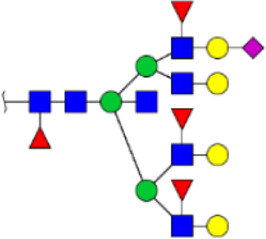	—	0.25
64	3491.297	3491.445	3491.345	[M + H]^+^	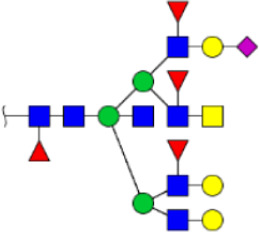	0.93	0.32
65	3650.300	3650.486	3491.452	[M + Na]^+^	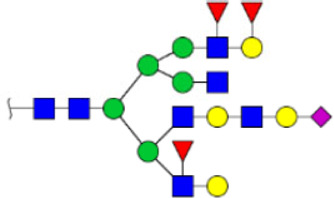	0.22	0.07
The N-glycan with GlcNAc, Galβ1-4GlcNAc moieties			Number			44	52
			Sum			63.20	79.04

^a^ Monosaccharides are represented according to MS tools from the GlycoWorkbench software. GlcNAc, blue square; Man, green circle; Gal, yellow circle; Fuc, red triangle; NeuAc, purple diamond.

^b^ \, not detected in the sample.

## Discussion

Despite the continuous improvement in medical technology and comprehensive treatment, the prognosis of ESCC patients remains poor (Yang et al., 2020a; Wei et al., 2020). It is reported that the overall survival rate of ESCC is as high as 20–30%, and there is still much room for improvement because the 5-year survival rate of ESCC patients could rapidly increase to 80–90% if they were detected in the early stage and received the timely intervention (Fitzmaurice et al., 2017; Hoshino et al., 2020). Unfortunately, since nearly half of early-stage ESCC patients were unlikely to show clinical symptoms, coupled with the lack of reliable noninvasive screening methods, more than 50% of ESCC patients were diagnosed at the advanced stages (Ohashi et al., 2015; Kojima and Doi, 2017). Therefore, to improve the prognosis of ESCC, it is still an urgent demand for the discovery of a novel noninvasive biomarker that can be detected even earlier.

Decades of research have demonstrated that aberrant protein glycosylation often occurs in the development of tumors, and it has been shown that specific tumor-associated glycans are expressed in the precursor lesions of different types of cancer, which makes them potentially powerful early diagnosis markers (Oliveira-Ferrer et al., 2017; Liu et al., 2018; Okumura et al., 2020; Schedin-Weiss et al., 2020; Zhang et al., 2020). Glycomic and glycoproteomic analysis of blood samples have shown that glycans or glycan profiles could be used as candidate biomarkers to distinguish EC from controls and potential predictors of disease progression (Yehia et al., 2009; Hammoud et al., 2010; Mohanty et al., 2012; Song et al., 2014). But the comprehensive information of salivary glycopatterns from ESCC and the possibility of salivary glycopatterns acting as potential biomarkers were not explicit yet.

**FIGURE 6:**
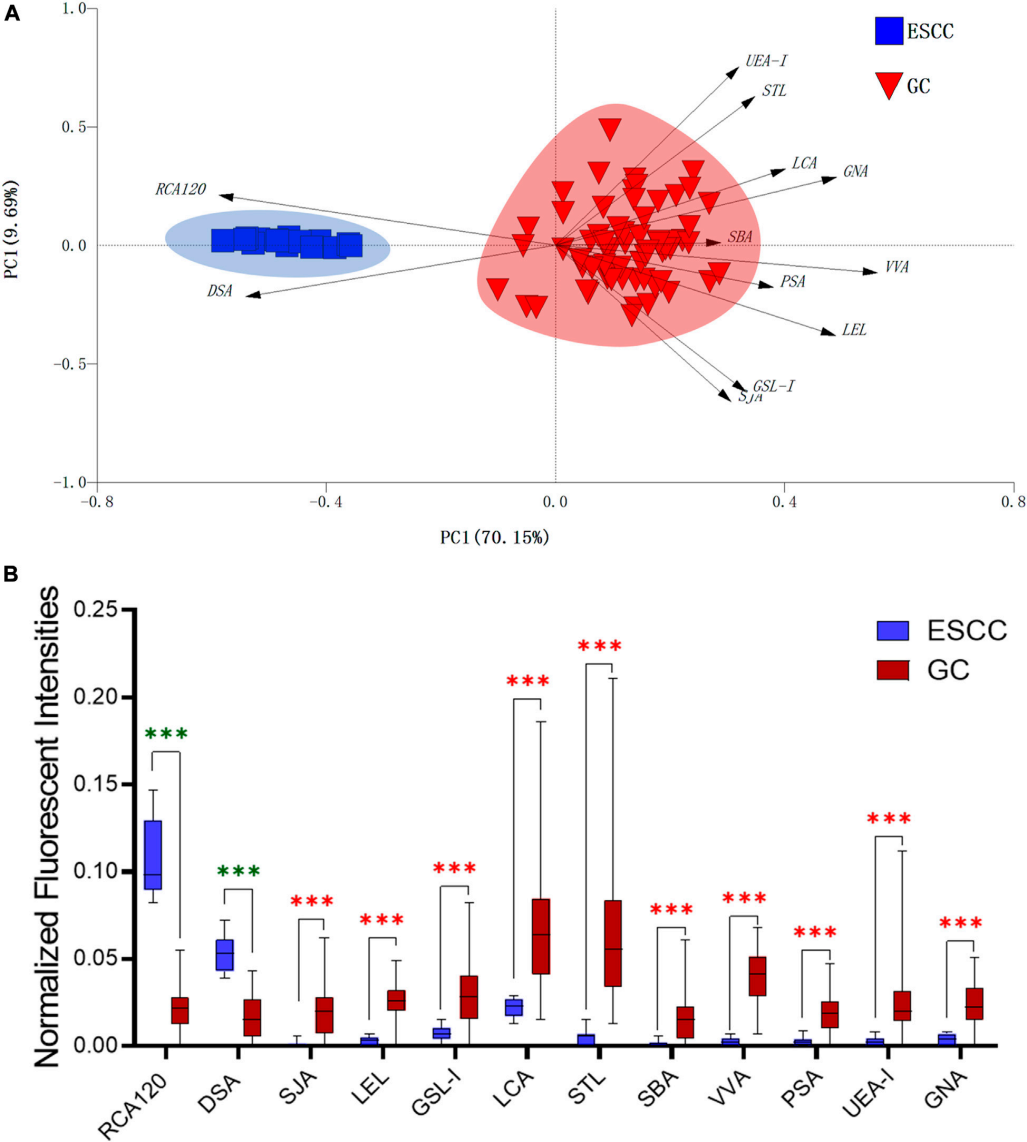
The different salivary glycopatterns in ESCC and GC patients. **(A)** The principal component analysis of the salivary glycopatterns from ESCC and GC patients is based on the NFIs of 37 lectins. ESCC and GC cases were indicated by blue squares and red triangles. **(B)** The alterations of salivary glycopatterns in ESCC patients compared to GC patients. The NFIs of two lectins (RCA120 and DSA) increased and 10 lectins decreased in ESCC patients compared to GC patients.

In this study, the integrated glycomics methods were used to investigate the differences of salivary glycopatterns between ESCC and HV cases. The results of lectin microarrays showed that 13 lectins (e.g., ECA, RCA120, and DSA) revealed significant alterations of the salivary glycopatterns between ESCC and HV cases. As a result, ESCC patients showed higher levels of GalNAc and Gal expression profile, sialic acid expression profile, GlcNAc expression profile, and lower levels of mannose expression profile and fucose expression profile. The lectins of DSA, ECA, LCA, PSA, and UEA-1 were randomly selected to confirm the differentially expressed sugar patterns. The lectin of DSA has an affinity to both GlcNAc and Galβ1-4GlcNAc, which can be used as an effective tool for the analysis of complex-type N-glycans. We used the DSA-magnetic particle conjugates to isolate the glycoproteins. And the glycan profiles of DSA-isolated glycoproteins were analyzed by MALDI-TOF/TOF-MS after liberation and purification. The results indicated that the proportion of the GlcNAc or Galβ1-4GlcNAc-containing *N-*glycans was increased in ESCC (79.04%) compared with HV (63.20%), which was consistent with the results of lectin microarrays. Compared with our previously published data of the other important gastrointestinal cancer, we found that the glycopatterns of ESCC and GC are obviously different (Figure 6A). The NFIs of RCA120 and DSA were higher in the saliva of ESCC patients, and the NFIs of SJA, LEL, GSL-I, LCA, STL, SBA, VVA, PSA, UEA-I, and GNA were higher in GC patients (Figure 6B). These results indicate that different tumors exhibit the different cancer-associated glycopatterns in saliva glycoproteins, but the detailed glycol-codes of cancers still need further characterization.

Our analyses have some limitations. First, it is a pilot study, the samples recruited are not enough, and an independent validation group is absent in this study. Second, the derivatization of sialic acids, such as permethylation and ethyl esterification, is needed for more accurate measurements of the released N-glycan profile. But our study provided comprehensive information of saliva glycopatterns from ESCC and the resolution of ESCC-associated glycopatterns that may contribute to understanding the complex physiological changes of ESCC patients. And the altered salivary glycopatterns such as GlcNAc or Galβ1-4GlcNAc-containing N-glycans recognized by DSA might be served as potential biomarkers for the diagnosis of ESCC patients.

## Data Availability

The original contributions presented in the study are included in the article/Supplementary Material; further inquiries can be directed to the corresponding authors.
